# Odour of domestic dogs infected with *Leishmania infantum* is attractive to female but not male sand flies: Evidence for parasite manipulation

**DOI:** 10.1371/journal.ppat.1009354

**Published:** 2021-03-18

**Authors:** Monica E. Staniek, James G. C. Hamilton

**Affiliations:** Division of Biomedical and Life Sciences, Faculty of Health and Medicine, Lancaster University, Lancashire, United Kingdom; Walter Reed Army Institute of Research, UNITED STATES

## Abstract

Globally visceral leishmaniasis (VL) causes thousands of human deaths every year. In South America, the etiologic agent, *Leishmania infantum*, is transmitted from an infected canine reservoir to human hosts by the bite of the sand fly vector; predominantly *Lutzomyia longipalpis*. Previous evidence from model rodent systems have suggested that the odour of infected hosts is altered by the parasite making them more attractive to the vector leading to an increased biting rate and improved transmission prospects for the pathogen. However, there has been no assessment of the effect of *Le infantum* infection on the attractiveness of dogs, which are the natural reservoirs for human infection. Hair collected from infected and uninfected dogs residing in a VL endemic city in Brazil was entrained to collect the volatile chemical odours present in the headspace. Female and male *Lu*. *longipalpis* sand flies were offered a choice of odour entrained from infected and uninfected dogs in a series of behavioural experiments. Odour of uninfected dogs was equally attractive to male or female *Lu*. *longipalpis* when compared to a solvent control. Female *Lu*. *longipalpis* were significantly more attracted to infected dog odour than uninfected dog odour in all 15 experimental replicates (average 45.7±0.87 females attracted to infected odour; 23.9±0.82 to uninfected odour; paired T-test, *P* = 0.000). Male *Lu*. *longipalpis* did not significantly prefer either infected or uninfected odour (average 36.1±0.4 males to infected odour; 35.7±0.6 to uninfected odour; paired T-test, *P* = 0.722). A significantly greater proportion of females chose the infected dog odour compared to the males (paired T-test, *P* = 0.000). The results showed that the odour of dogs infected with *Le*. *infantum* was significantly more attractive to blood-seeking female sand flies than it was to male sand flies. This is strong evidence for parasite manipulation of the host odour in a natural transmission system and indicates that infected dogs may have a disproportionate significance in maintaining infection in the canine and human population.

## Author summary

Visceral leishmaniasis (VL) is a disease caused by the Protist parasite *Leishmania infantum*. In Brazil and other South and Central American countries, the parasite is transmitted by the bite of infected female *Lutzomyia longipalpis* sand flies. The disease leads to thousands of human deaths every year. Domestic dogs are the reservoir of infection therefore understanding how the infection affects the attractiveness of dogs to the insect vector is important in understanding the epidemiology of the disease. Golden Hamsters infected with *Le*. *infantum* are more attractive than uninfected hamsters to *Lu*. *longipalpis* however, it is not known if the attractiveness of infected dogs is similarly altered. In this study we showed that the odour of infected dogs is significantly more attractive to female sand flies, that can transmit the pathogen, than it is to male sand flies which can not. This clear-cut difference in attraction of female and males suggests that the females are preferentially attracted by parasite infected hosts and may lead to enhanced infection and transmission opportunities for the parasite.

## Introduction

In South America the protist *Leishmania (Le*.*) infantum* (Cunha & Chagas) (Kinetoplastida: Trypanosomatidae) causes the disease visceral leishmaniasis (VL) in humans and dogs (*Canis lupus familiaris*) [1]. The intracellular parasite, which is a pathogen of the immune system, primarily affects the reticuloendothelial system, surviving and multiplying in macrophage and dendritic cells [2]. In humans, VL is characterized by prolonged irregular fever, splenomegaly, hepatomegaly, pancytopenia and weight loss and in the absence of treatment the case fatality rate is >90% [3].

Globally, approximately 50,000 to 90,000 new cases of VL occur each year with more than 95% of them occurring in ten countries: Brazil, China, Ethiopia, India, Iraq, Kenya, Nepal, Somalia, South Sudan and Sudan [4]. In the Americas, VL is a significant public health problem due to its’ morbidity, mortality and broad geographical distribution [5]. From 2001 to 2015, 52,176 cases of visceral leishmaniasis were registered in the Americas, with 96.4% of these cases (50,268) reported from Brazil [5,6]. Overall, the burden of the disease (age-standardized DALYs) in Brazil has almost doubled during the period from 1990 to 2016 [7].

In Brazil *Leishmania infantum* infection is a zoonosis, with canines as the primary reservoir hosts. Humans, which are considered to be a dead-end host for the parasite [8], become infected when they are fed on by infected female sand flies of the *Lutzomyia longipalpis s*.*l*. (Lutz & Neiva) (Diptera: Psychodidae) species complex predominantly [9]. In dogs, VL is characterised by dermatitis, lymphadenomegaly, general muscular atrophy, and renal disease [10].

Host seeking haematophagous insects orientate towards and identify potential host animals by combinations of visual, thermal, tactile and chemical (host odour) cues produced by the host [11]. Together these stimuli, along with the individual response of female insects, dictate the likelihood that a haematophagous insect will successfully obtain a blood meal [11–13]. For temporary ectoparasites (e.g. mosquitoes, triatomines, midges and sand flies) host odour is of primary importance in host location. Parasite manipulation of the host can enhance transmission opportunities in favour of the parasite. There is now significant evidence that parasite infection of the host animal alters both the vector host choice behaviour as well as vector biting rate and blood-meal size [14]. The choice to bite an infected or uninfected host is key for pathogen transmission and numerous empirical studies show that pathogens can modify the scent of infected hosts to attract vectors [15].

Reports have shown that mosquitoes are more attracted to hosts (including humans, mice and birds) infected with malaria than uninfected hosts [16–20]. Humans infected with *Plasmodium falciparum* are more attractive to *Anopheles gambiae s*.*l*. than uninfected humans. The increased attraction is most pronounced when the parasite is in the most transmissible (gametocyte) stage [21] and the increased host parasitaemia may partially, at least, be induced by mosquito biting activity which leads to higher transmission rates [22]. The difference in attractiveness has been related to the presence of a parasite produced isoprenoid precursor, (*E*)-4-hydroxy-3-methyl-but-2-enyl pyrophosphate (HMBPP) in host blood which has been shown to trigger an enhanced release of attractants which increases the likelihood of an infected human host being bitten by the vector [23].

In contrast only a limited number of studies have been carried out to determine if *Leishmania* parasites manipulate host odour. In olfactometer choice experiments volatile odours collected from hamsters infected with *Le*. *infantum* (MHOM/BR/74/PP75) were found to be more attractive to female *Lu*. *longipalpis* sand flies than uninfected hamsters [24]. A subsequent laboratory study investigated the change in attractiveness of individual hamsters infected with *Leishmania infantum* and showed that the odour of a significant number of hamsters became significantly more attractive to the vector 122±8.9 days after infection [25], potentially after the parasite skin load had increased [8,26]. A coupled gas chromatography-mass spectrometry (GC/MS) and statistical analysis (principal component analysis, PCA) compared the solid phase microextraction (SPME) entrained odours of infected and uninfected Brazilian dogs and identified several markers of infection [27,28]. Some of these chemicals, when tested at high concentrations (undiluted or diluted 50%), were found to activate female *Lu*. *longipalpis* and to activate and attract male *Lu*. *longipalpis* [29].

None of these previous studies have conclusively demonstrated that the odour of *Le*. *infantum* infected dogs, the natural reservoir host, is more attractive to the sand fly vector. The experiments with hamsters were carried out with a non-natural host/parasite system and the experiments with canine odour, although showing a difference in odour profile between infected and uninfected dogs, did not test if the infected dog odour was more or less attractive than the uninfected dog odour.

We therefore carried out a study on the odours collected from the hair of infected and uninfected dogs in a Y-tube choice olfactometer, to determine if female or male *Lu*. *longipalpis* had a preference for infected dog odour.

## Results

### Female sand fly response to infected and uninfected dog odour

The data were found to be normally distributed (Ryan-Joiner test, *P*>0.1)

**Fig 1:**
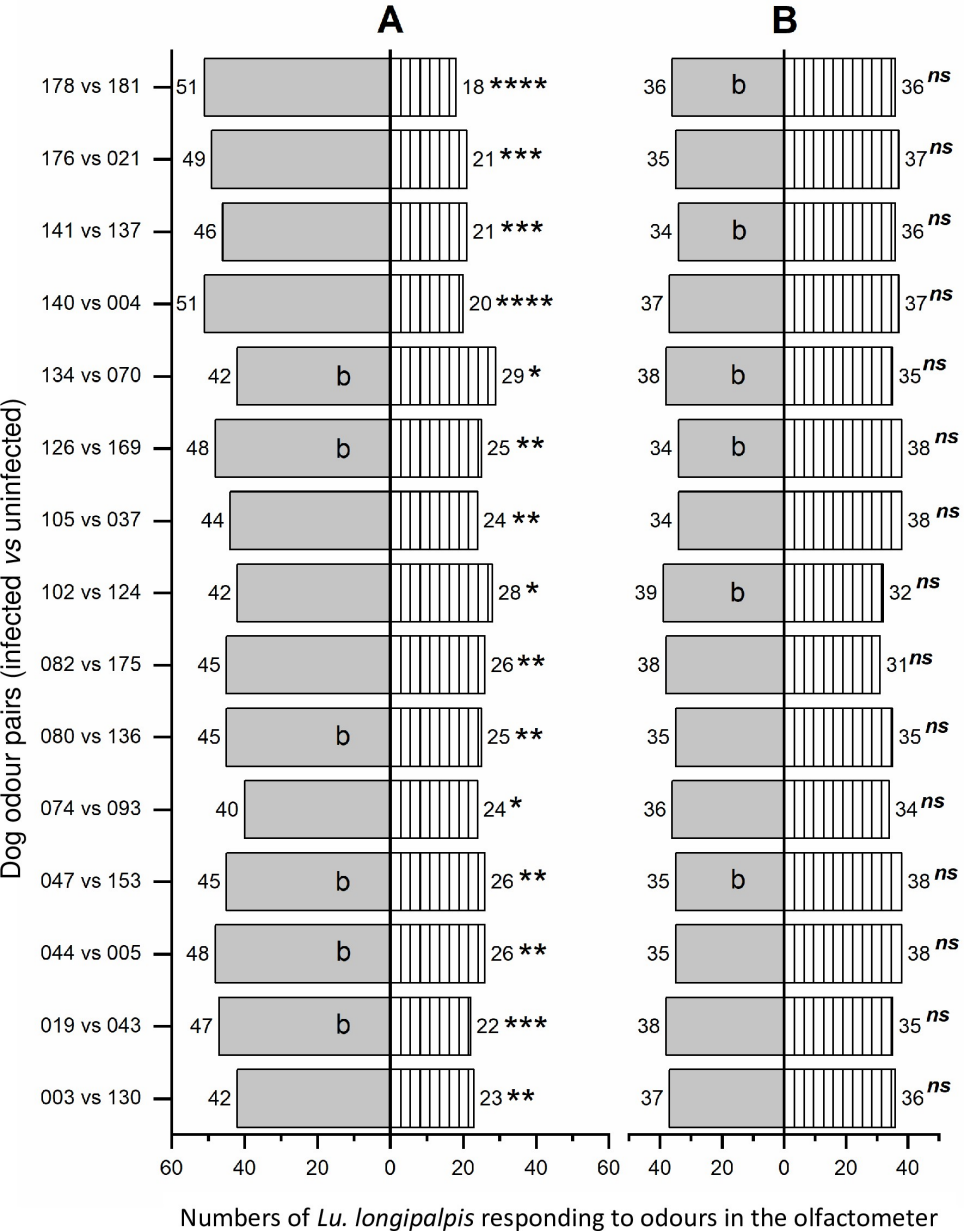
Numbers of female (A) and male (B) *Lutzomyia longipalpis* attracted to volatiles collected from infected and uninfected dogs.

In all 15 individual experimental replicates, female *Lu*. *longipalpis* were significantly attracted to the infected dog odour compared to the uninfected odour (Fig 1A and S1 Table).

We combined the data from the blinded and unblinded experiments for each of the infected and uninfected data sets as there was no significant difference between them [infected dog odour; unblinded 45.6±1.3 *vs* blinded 45.83±1.0 (2-sample T-test, *P* = 0.869); uninfected dog odour; unblinded 22.8±1.1 *vs* blinded 25.5±1.0 (2-sample T-test; *P* = 0.0874)] and then compared the response to each (mean response to infected odour 45.7±0.87; mean response to uninfected odour 23.9±0.82). Female sand flies were significantly attracted to the infected odour (paired T-test, *P* = 0.000).

The numbers of female (panel A) or male (panel B) *Lu*. *longipalpis* attracted to the infected dog odour (grey bars) or uninfected dog odour (hatched bars) in the Y-tube olfactometer. The dog odour pairs are indicated on the y-axis, the first in each pair is from an infected dog and the 2^nd^ is from an uninfected animal. The numbers of sand flies attracted to each side of the olfactometer are given beside each bar. The “b” in some bars indicates that the experiment was blinded as the dog infection status was unknown to the experimenter. Statistical significance was analysed by binomial test comparing the actual number of sand flies that responded to infected odour compared to the number that responded to the uninfected odours (*P≤0.05, **P≤0.01, ***P≤0.001, ****P≤0.0001, ns = not significant).

### Male sand fly response to infected and uninfected dog odour

In all 15 of the individual experimental replicates, male *Lu*. *longipalpis* were not significantly more attracted to the infected dog odour entrainment extracts compared to the uninfected dog odour (P>0.05) (Fig 1B and S2 Table).

We combined the data from the blinded and unblinded experiments for each of the infected and uninfected data sets as there was no significant difference between them [infected dog odour; unblinded 36.1±0.5 vs blinded 36.0±0.9 (2-sample T-test; *P* = 0.913); uninfected dog odour; unblinded 35.8±0.8 vs blinded 35.8±0.9 (2-sample T-test; *P* = 0.890)] and then compared response to each (mean response to infected odour 36.1±0.4; mean response to uninfected odour 35.7±0.6). Male sand flies were equally attracted to the infected and uninfected odours (paired T-test; *P* = 0.722).

### Comparison of female and male response to infected dog odour

On average significantly more of the females that responded (65.7±1.1%) were attracted to the infected dog odour than the males 50.3±0.6% of the males (T-test; *P*<0.000).

### Relationship between parasite load, discriminate value and the female and male sand fly response

There was no significant relationship between the parasite load and the proportion of females (or males) that were attracted to the infected dog hair odour [Regression analysis: females R^2^(adjusted) = 0.00%; males R^2^(adjusted) = 0.00%]. There was a slight but non-significant relationship between the previously identified discriminate values [30] and the proportion of females (or males) that were attracted to the infected dog hair odour [Regression analysis: females: R^2^(adjusted = 8.1%); males R^2^(adjusted) = 16.6%, *P* = 0.074]. A correlation analysis suggested a negative relationship between the discrimination value of infected dog odour and male response (Pearson r = -0.25). Removal of the parasite load value of 853.4 parasites ml^-1^, which is a potential outlier data point, had little effect on R^2^(adjusted) for either the female or male response to parasite load however the relationship between discrimination factor became slightly more important for the female sand fly response [R^2^(adjusted) = 8.9% P = 0.158] and for the males [R^2^(adjusted) = 9.4% P = 0.152]. Correlation analysis suggested a moderate relationship between the discriminate value and female response (Pearson r = 0.4) and male response (Pearson r = -0.4).

### Comparison of the attractiveness of uninfected dog odour versus hexane to female and male sand flies

Both female and male sand flies were significantly attracted to dog odour when given the choice of uninfected dog odour (in hexane solvent) or hexane solvent only. A significantly greater proportion of female sand flies (66.5%) were attracted to the uninfected dog volatiles than to hexane solvent only (Binomial test; P = 0.000) and of the male sand flies that responded, 62.9% were attracted to uninfected dog odour compared to hexane solvent only (Binomial test; P = 0.000) (Table 1).

**Table 1:** Response of female and male *Lutzomyia longipalpis* to odour of uninfected dogs and hexane only in Y-tube olfactometer choice experiments.

female sand fly response					
**expt**	**dog i.d.**	**uninfected dog odour**	**hexane**	**no-response**	***P***
**1**	138	48	21	11	***
**2**	128	45	29	6	*
**3**	027	46	20	14	***
	cumulative	139	70	31	****
male sand fly response					
**expt**	**dog i.d.**	**uninfected dog odour**	**hexane**	**no-response**	***P***
**4**	138	43	27	10	*
**5**	128	47	22	11	***
**6**	027	42	29	9	*
	cumulative	132	78	30	****

Female and male sand fly response = control experiments measuring either the response of the female or male sand flies in the Y-tube olfactometer; expt = experimental replicate; dog i.d. = dog identification number; uninfected dog odour = number of sand flies attracted to the dog odour sample; hexane = number of sand flies attracted to hexane only; no-response = number of sand flies that did not respond. Statistical significance was analysed by binomial test comparison of number of sand flies responding to the dog odour vs hexane (*P≤0.05, ***P≤0.001, ****P≤0.0001).

## Discussion

Domestic dogs, *Canis lupus familiaris*, are the primary reservoir host of the protist parasite *Le*. *infantum* which is transmitted in Brazil and other South American countries by the blood-feeding activity of female *Lu*. *longipalpis* sand flies. This study has shown that even though dog odour was attractive to both female and male sand flies, the odour of dogs naturally infected with *Le*. *infantum* was significantly more attractive to female *Lu*. *longipalpis* than the odour of uninfected dogs. In olfactometer choice experiments more female sand flies (mean±sem, 45.7±0.9) were attracted to infected dog odour than were attracted to uninfected (23.9±0.8) dog odour. By comparison male *Lu*. *longipalpis* did not differentiate between infected (36.1±0.4) and uninfected (35.7±0.6) dog odour. In both sets of experiments female and male *Lu*. *longipalpis* were highly attracted to dog odour with only 13 and 10% respectively failing to respond to the dog odour in the olfactometer.

Previous studies using model infection systems have indicated that the odour of host animals infected with *Le*. *infantum* was more attractive to the sand fly *Lu*. *longipalpis*. A study with Golden Hamsters showed that the odour of hamsters infected with *Le*. *infantum* was more attractive than odour from uninfected hamsters [24] and a subsequent study showed that the odour of 6 out of 13 Golden hamsters infected with *Le*. *infantum* became significantly more attractive to the majority of *Lu*. *longipalpis* females after a prolonged period of infection [25]. Both of these studies used Golden Hamsters which are not naturally exposed to blood-feeding by *Lu*. *longipalpis* or infection with *Le*. *infantum* in the wild. In that study *Le*. *infantum* infected Golden Hamsters were not attractive to males. This is the first occasion, that we are aware of, when *Lu*. *longipalpis* females and males have been offered the choice of entrained headspace volatile odours from infected and uninfected dogs. The results are significant because in South and Central American as well as Mediterranean countries, dogs are the reservoir of infection and are essential to the maintenance of the zoonotic transmission cycle that leads to human infection [31]. The results of this study differ in 2 important ways from those obtained from previous work using infected Golden Hamster odour [25]. First, in this study all of the infected dogs were more attractive to female sand flies than uninfected dogs whereas by comparison only half of the infected hamsters were more attractive than uninfected hamsters to sand flies. This suggests that infection had a greater effect on dog odour than hamster odour and thus the sand fly response to dog odour compared to hamster odour. Second, in this study 90% of male *Lu*. *longipalpis* were attracted to dog odour whereas approximately only 50% of male sand flies were attracted to hamster odour. These results highlight the potential limitations of using model rather than real systems to explore pathogenic parasite host vector interactions.

In a series of analytical chemistry studies, the odour of dogs infected with *Le*. *infantum* were shown to have a different odour profile to uninfected dogs [28]. Subsequently, 4 compounds identified as potential biomarkers of infection [32] were tested in a wind-tunnel olfactometer at either 50% or 100% concentration. In that study some of the compounds were found to activate upwind flight and to be marginally attractive to males. Female sand flies were activated but not attracted by the marker compounds [29]. The responses of both male and female *Lu*. *longipalpis* to these synthetic compounds were substantially lower than the responses seen in this study. It is not possible to be sure if the observed differences are because of differences in; olfactometer design, sample size, the range of different compounds and concentrations presented to the sand flies, the method of preparing the dog odour extracts (or a combination of some or all of these factors) all of which differed substantially between the two studies.

Although both male and female *Lu*. *longipalpis* were attracted to uninfected dog odour only female sand flies showed enhanced attraction to infected dog odour. The differences between the female and male sand fly response to infected dog odour observed in this study may also be important to transmission of the parasite. *Lutzomyia longipalpis* mating behaviour is believed to be driven by male lekking behaviour in which male *Lu*. *longipalpis* first locate the host and establish mating aggregations [33] where they compete with each other for territory, produce a sex/aggregation pheromone and females are attracted by a combination of the host odour and pheromone [34–36]. When the females arrive at the lekking site, they choose a mate, take a blood-meal and depart [37]. In this model, males first select the host animal and the females arrive later [34,35,38]. The current study suggests a more nuanced situation where females can choose host animals independently of their need for associated male pheromone and it is this behaviour that can be manipulated by the parasite. The majority of sand fly species do not lek and therefore it might be possible for female *Lu*. *longipalpis* to also locate a host and take a blood meal without being attracted to a lek site. By not attracting male *Lu*. *longipalpis* to infected dogs, male aggregations would be more likely to take place on uninfected dogs (as they would represent the majority of the canine population). Thus, we might expect infected female sand flies to be more likely to feed on uninfected dogs on which leks had already been established and thereby satisfying the proposed need [14] of infected vectors to feed on uninfected hosts to maintain disease transmission. To test this hypothesis it will be important to determine if sex pheromone combined with uninfected dog odour is more or less attractive than infected dog odour by itself to infected sand flies.

Our results showed that the parasite load, measured in the blood of infected dogs, was not significantly related to the attraction of female *Lu*. *longipalpis* to infected dogs. Parasite load, measured in both blood and skin, has been shown to be related to infectiousness of the dog to the vector, although parasite load in blood or skin is not related to symptoms of infection [39]. In the future it would be interesting to determine if sand fly attraction was related to skin parasite load.

There was a slight positive correlation between odours that were more attractive to females and the VOC analyser discrimination response (obtained from a previous study [30]) and a slightly greater negative correlation in the male response. This suggests that the sand flies and VOC analyser are detecting similar odour molecules. A negative correlation between male sand fly response and VOC analyser discrimination response might suggest that dogs with increasingly atypical odours are avoided by male sand flies. It may also suggest that specific odour components such as CO_2_ and other highly volatile breath odour components, which would be missing in the extracts used in this study have differential importance as host cues.

In the future it will be important to determine if this observed phenomenon also occurs with live infected and infectious dogs and wild *Lu*. *longipalpis* in the peridomestic environment where a number of other factors (e.g. odour of competing alternative animal hosts, the presence of sex/aggregation pheromone and plant odours) might contribute to divert or dilute the response of the female sand flies to infected dog odour. In addition, synthetic sex/aggregation pheromone is a strong attractant [40,41] that can be used to disrupt aggregation behaviour at normal lekking sites for example in chicken sheds [42]. In the future it will be important to determine if the synthetic pheromone is also able to compete with and disrupt the enhanced attractiveness of infected dogs as this is an additional mechanism by which normal transmission of *Le*. *infantum* might be reduced [43].

In this study the maximum distance that either a male or female sand fly could travel is 20 cm. A Y-tube olfactometer is useful for determining if a choice is made between one odour or another. It would be interesting to determine the response of *Lu*. *longipalpis* to the odours tested in this study over longer distance in a wind-tunnel [29] or by using mark-release-recapture experiments in a natural setting over significantly greater distances [42] to potentially reveal in more detail responses to and behaviour elicited by infected and uninfected odours.

It has been suggested that human odour-baited traps combined with insecticides could improve capture and kill of mosquito vectors [44–46] or cattle or pig odour could be useful for control of teste flies [47]. The observed sex specific increase in attraction to infected dogs, as well as the work of others [29], suggests that infected dog odour may also provide geographically generic opportunities to develop new vector control methods. Clearly control devices which successfully target females are much more likely to have a significant effect on disease transmission than devices that target males. However, further studies are required to identify any new compounds, in addition to those previously identified, as well as formulate and evaluate them in appropriate strategies [48]. In addition, combining host odour kairomones along with synthetic sex/aggregation pheromone could lead to greater opportunities to reduce *Leishmania* parasite transmission [41,43].

In this study we have shown that the odour of *Leishmania infantum* infected dogs is more attractive to female *Lu*. *longipalpis* than the odour of uninfected dogs. We have also shown that male *Lu*. *longipalpis* do not exhibit this enhanced attraction. It is proposed that this is a clear example of parasite manipulation of the host: first in that odour of infected dogs is more attractive than uninfected dogs and second female sand flies are affected by the changed odour but not males. However, carefully controlled experiments with infected dogs in a natural setting should be carried out to confirm and extend these observations and to determine any likely impact on *Leishmania* transmission and thus epidemiology of the disease. *Leishmania infantum* is considered by many to have been relatively recently introduced to the New World [49] and as such the evolutionary relationship between parasite and insect vector (itself an incipient species complex [50]) is relatively new. Thus, it will be interesting to determine if the observed parasite-host-vector relationship is also true in the Old World *Leishmania infantum*-*Canis familiaris*-*Phlebotomus perniciosus* relationship.

## Materials and methods

### Ethics statement

Dog blood and hair samples were taken from dogs that were also microchipped with the informed consent of their owners. Ethical approval was obtained from the Comissão de Ética no Uso de Animais (CEUA), Instituto Oswaldo Cruz (licence L-027/2017) in Brazil and Lancaster University Animal Welfare and Ethics Review Board (AWERB) in the UK. The CEUA approval complied with the provisions of Brazilian Law 11794/08, which provides for the scientific use of animals, including the principles of the Sociedade Brasileira de Ciência em Animais de Laboratório (SBCAL). The AWERB approval complied with the UK Home Office guidelines of the Animals in Science Regulation Unit (ASRU) and complied with the Animals (Scientific Procedures) Act (ASPA) 1986 (amended 2012) regulations and was consistent with UK Animal Welfare Act 2006.

### Field study site, dog recruitment and sample collection

The study was carried out on the entrained odour of hair collected from infected and uninfected dogs recruited in April 2018 in Governador Valadares (GV), a municipality of approximately 280,000 people in Minas Gerais State, Brazil (18°51’12”S W; 41°56’42”W, altitude 170m) 320 km northeast of Belo Horizonte, the state capital.

GV is situated in the Rio Doce basin within the Atlantic Forest region the local topography consists of valleys and hills. The climate is Aw (tropical sub-warm and sub-dry) according to the Köppen–Geiger classification [51]. The city has an average temperature of 24.2°C (annual range 15.2–33°C) and average annual rainfall, concentrated between October and March, of 1109 mm [52].

The area is a focus of intense VL transmission and is also endemic for cutaneous leishmaniasis where the sand fly vector, *Lu*. *longipalpis* is abundant [53,54]. In GV 212 cases of human VL were reported between 2008–2015, the cumulative VL incidence was 7 cases per 100,000 [55] with case fatality rate estimates of 8.9%-16.3% [56,57]. In 2017, just before this study, case fatality rate was 6.4 per 100,000 [58]. Canine infection reported for the 2008–2012 period was 29% (8,622/29,724) in GV [59].

Recruitment and sampling of 133 dogs was carried out in the Altinópolos district of Governador Valadares which was chosen because of the high prevalence of canine VL (cVL) (average incidence reported in 2013 was 33.8%) [53] and the presence of a large population of household-owned dogs (n = approx. 2000) (Centro de Controle de Zoonoses (CCZ) survey). The recruitment of domestic dogs and collection of individual hair and blood samples was undertaken with the informed consent of the dog’s owners [30]. Briefly, dogs were chosen at random by walking through the area and visually identifying potential recruits and then seeking owner consent. Inclusion criteria were dogs aged ≥3 months, without previous clinical assessment or laboratory diagnosis for cVL. Exclusion criteria were pregnant/lactating bitches, aggressive or stray dogs. Canine dorsal hair and blood samples were obtained as previously described [30].

### Diagnosis of dogs and parasite load calculation

The diagnosis of each dog was performed previously as reported [30]. Briefly, DNA was extracted from the blood sample of each individual dog and a PCR was performed in triplicate using the primer pair Mary F and Mary R [60]. Diagnosis was based on the outcomes of three PCR replicates of each dog DNA sample. Parasite load was calculated by qPCR also using the primer pair Mary F and Mary R and a standard curve was established using extracted *Le*. *infantum* DNA; 1:10 serial dilutions, ranging from 0.01 to 10,000 parasites per ml and used to quantify the number of parasites in the dog blood samples [30].

### Entrainment of volatile organic compounds

Volatile organic chemicals (VOCs) from all the dog hair samples were entrained as described previously [61]. Briefly, charcoal-filtered air (15ml sec^-1^) was passed through the inlet port of a quick-fit Dreschel head into a 50ml round-bottom quick-fit glass flask which contained the dog hair (1g). The air from the effluent port of the Dreschel head passed into a Orbo-402 Tenax-TA trap (8.75cm) (Sigma-Aldrich Company Ltd, Dorset, UK) via a 10cm length of Teflon tubing. Hair samples were entrained for 2.5h and the collected VOCs were recovered by elution of the Orbo tube with *n*-hexane (SupraSolv grade; Merck, Germany) (2ml). A fresh Orbo tube was used for each entrainment dog hair sample. Each eluted sample was concentrated to a final volume (ca. 500μl) under nitrogen and then sealed in a clean glass Pasteur pipette and stored (-20°C) until further analysis.

### Bioassay

#### Sand flies

The sand flies used in the bioassay were from a colony maintained at Lancaster University that was originally established from individuals collected in Jacobina, Brazil (40°31′ W, 11°11′S). Flies were reared according to the method of Lawyer *et al* [62] and adults maintained in Nylon Barraud cages (18 x 18 x 18 cm) at 27°C (range 25–29°C), 95% RH with a 12:12 h (L:D) photoperiod. Female sand flies were fed sheep blood (TCS Biosciences Ltd, Buckingham, UK) via a chicken skin membrane before egg laying [63–65]. To obtain virgin female and male *Lu*. *longipalpis* for use in the bioassays newly eclosed adults were separated within 6h of emergence (before rotation of external male genitalia) and fed on saturated sucrose solution only. They were then held in a Barraud cage, kept inside a plastic bag to maintain humidity at 27°C with access to sugar (*ad libidum*) in the insectary for 5 to 7 days until used in the bioassay.

Bioassays were performed as previously described [61]. One hour prior to the start of the experiment the sand fly holding cage was moved into the bioassay room where the plastic bag was removed and the sand flies allowed to acclimatise to the room conditions (temp; mean 27°C, range 25–29°C: humidity; mean 75%, range 68–78%). All bioassays were performed at the same time of day (12:00–15:00 hr) to minimise any potential effect of circadian rhythms on flight activity [66].

#### Y-tube olfactometer

The Y-tube olfactometer and associated Teflon tubing was designed and assembled were as previously described [36,61]. The maximum distance which a sand fly could travel if attracted was 20 cm. This apparatus allowed us to enter the entrained dog odour solutions into the air flowing through the Teflon tubing connected to the olfactometer arm. The solution was injected onto a rolled up filter paper disk (Grade 1; 20mm diam., Whatman International Ltd., Maidstone, United Kingdom) via a small hole drilled in the wall of the tubing. A flow of clean air (ZeroGrade; BOC, Lancaster, UK) through the apparatus was controlled by a two-stage cylinder regulator valve (BOC Series 8500 Air Regulator BOC) and a rotameter (Sigma-Aldrich Company Ltd, UK), adjusted to 5ml sec^-1^ and periodically confirmed using a bubble meter. All connections between Teflon tubing components were made airtight by sealing with Teflon tape (Sigma-Aldrich Company Ltd, UK). Glass wool was inserted into the end of each Y-tube olfactometer arm to prevent flies from escaping into the apparatus.

All Teflon tubing and glassware was cleaned 24h prior use. Glassware was washed with 10% Teepol solution, distilled water and then acetone before being baked overnight at 225°C. Teflon tubing was rinsed with hexane (Pesticide Grade, Fisher Scientific UK Ltd, Loughborough, UK) and allowed to air dry overnight.

To perform the bioassay, the Y-tube was placed horizontally on a solid bench, 1μl of an infected dog odour extract was injected onto the filter paper in one side of the apparatus and 1μl of an uninfected dog odour extract for comparison was injected into the other side of the apparatus. The holes in the wall of the Teflon tubes were sealed with PTFE tape.

To test the response of sand flies, each fly was removed from the holding cage with an aspirator and released at the open end of the stem of the Y-tube olfactometer. A timer was started and after three minutes the position of the sand fly within the Y-tube was recorded; either the test or control arm or, if it remained in the stem, a “no choice” was recorded. After 10 replicates, the rolled-up filter paper was removed and replaced, and to reduce any potential spatial bias in the apparatus, the position of the test and control arms were swapped round by rotating the olfactometer through 180^o^ in the horizontal plane. In total 80 female or 80 male sand flies were used per experimental replicate (i.e. each pair of infected and uninfected dog odour combinations were tested with 80 sand flies).

#### Dog sample selection

From the pool of available (n = 133) dog hair odour samples, we selected the extracts collected from the hair of 15 infected and 15 uninfected dogs (n = 30). Selection of the infected and uninfected dog hair odour extracts was based on PCR and qPCR analysis results. The selected infected dog hair extracts were from dogs with a parasite load that ranged from 1.3 ml^-1^ to 853.4 parasites ml^-1^ of blood. The uninfected dog hair extracts were from dogs with no detectable parasite DNA. Eleven of the selected infected dogs were asymptomatic, 2 were oligosymptomatic (between 1 and 3 symptoms) and 2 were symptomatic (more than 3 symptoms) (S3 Table).

To determine if any attraction observed in the Y-tube experiments might be related to the discrimination value previously obtained from a VOC analyser (eNose) study [30] the 15 infected and 15 uninfected dog hair odour samples were also selected according to their discrimination values. The VOC analyser data analysis differentiated between the volatile odour of each dog and the other dog odours based on a dog’s odour cluster probability (discrimination factor). Those dogs that gave a VOC analyser response that was very different from other dogs were classed as “high discrimination”. Other odours that were not as well differentiated were classed as “medium discrimination” and those that did not show much differentiation were classified as “low discrimination”. We selected 5 high, 5 medium and 5 low discriminated individuals to include in each set of 15 infected and 15 uninfected dog hair samples for comparison. The pairings are summarised in S4 Table.

### Study design

The response of female (or male sand flies) to each infected dog hair odour sample was compared with their response to an uninfected dog hair sample in the Y-tube olfactometer. High, medium and low discriminated infected samples were cross compared with either a high, medium or low discriminated uninfected sample. The same 15 pairs of dog hair odour were used in both the female and male bioassays, although in a different order (S1 and S2 Tables).

The 15 infected and uninfected dog odour pairs were divided into 2 sub-groups. The infection status of the first group of 9 pairs of dog odour samples was known (unblinded). The infection status of the second subgroup of 6 dogs was hidden, to avoid any potential experimental bias (blinded).

All dog odour extracts were diluted by a factor of 1:10 to achieve optimal sand fly response [67]. Control bioassays were performed to determine female and male response to uninfected dog hair odour extract compared to a hexane control.

### Data analysis

Normality tests (Ryan-Joiner, similar to Shapiro-Wilk) confirmed that data were normally distributed.

Binomial tests [68] were used to determine whether a greater number of female or male sand flies were attracted to infected dog odour extract than uninfected dog odour extract than would be expected by chance (50/50) for each experimental replicate. A binomial test was also used to compare response of female and male *Lu*. *longipalpis* to uninfected dog odour.

Response of female or male sand flies to infected or uninfected dog odour in the unblinded and blinded experiments was compared using two-tailed T-tests.

Response of female and male sand flies to the infected dog odour or uninfected dog odour was compared using two-tailed paired T-tests.

Response of female sand flies to infected dog odour extract was compared to response of male sand flies to infected dog odour extract using a two-tailed T-test.

Control data were analysed by binomial test.

To determine if there was a relationship between parasite load and the female and male sand fly response, we carried out regression analyses on the proportion of females (and males) that were attracted to the infected dog hair odour and the parasite load of the dog. A correlation analysis was used to determine if there was a relationship between the discrimination status of odour extracts obtained in the VOC analyser study and the proportion of female or male sand flies that responded to the extracts.

All statistical analyses were performed using MINITAB 19 (Minitab, Inc. 2020).

## Supporting information

S1 Table Female *Lutzomyia longipalpis* response to entrained odour of infected and uninfected dogs in unblinded and blinded Y-tube olfactometer experiments. Summary of blinded and unblinded Y-tube bioassay experiments for female *Lu*. *longipalpis*. The odour extracts from infected dogs (in bold) and uninfected dogs are shown. Infected = the number of male sand flies attracted to the arm of the Y-tube containing infected odour; uninfected = the number of male sand flies attracted to the arm of the Y-tube containing uninfected odour; no-response = number of sand flies that did not respond in the Y-tube experiment. a = unblinded experimental replicates, b = blinded experimental replicates. Statistical significance was analysed by binomial test comparison of number of sand flies responding to test and control odours (*P≤0.05, **P≤0.01, ***P≤0.001, ****P≤0.0001).
(DOCX)

S2 Table Male *Lutzomyia longipalpis* response to entrained odour of infected and uninfected dogs in the unblinded and blinded Y-tube olfactometer experiments. Summary of blinded and unblinded Y-tube bioassay experiments for male *Lu*. *longipalpis*. The odour extracts from infected dogs (in bold) and uninfected dogs are shown. Infected = the number of male sand flies attracted to the arm of the Y-tube containing infected odour; uninfected = the number of male sand flies attracted to the arm of the Y-tube containing uninfected odour; no-response = number of sand flies that did not respond in the Y-tube experiment. a = unblinded experimental replicates, b = blinded experimental replicates. Statistical significance was analysed by binomial test comparison of number of sand flies responding to test and control odours (*ns* = not significant). The experimental outcomes are presented in the same order as for the female bioassays (S1 Table) for ease of comparison.
(DOCX)

S3 Table Dog identification number, infection status, symptoms and parasite load of the infected dogs from which the entrained odour used in the Y-tube olfactometer bioassay was obtained. Dogs were classified as asymptomatic (the absence of clinical signs), oligosymptomatic (the presence of one to three clinical signs) and symptomatic (the presence of more than three clinical signs according to Mancianti et al. [69]. Infection category and symptoms were assessed by Governador Valadares CCZ clinical staff. Parasite load (number of parasites ml^-1^) was assessed by qPCR as previously described [30].
(DOCX)

S4 Table Discrimination status of infected and uninfected dog hair odours used in the Y-tube olfactometer experiments with female and male *Lutzomyia longipalpis*. Dog hair odour pairings: the infected and uninfected dog hair odour samples which were compared are shown along with their discrimination values. VOC analyser discrimination value: the VOC analyser discrimination status of each dog is shown. *Leishmania infantum* infected dogs are denoted in bold text. The infection status of samples 1–9 was known to the experimenter and samples 10–15 were unknown (blinded).
(DOCX)
