# Supplementary material for: Odour of domestic dogs infected with Leishmania infantum is attractive to female but not male sand flies: Evidence for parasite manipulation
Source: PLoS Pathog. 2021 Mar 18;17(3):e1009354. doi: 10.1371/journal.ppat.1009354 (PMC7971543; doi:10.1371/journal.ppat.1009354)
Supplement: S2 Table — Summary of blinded and unblinded Y-tube bioassay experiments for male Lu. longipalpis. The odour extracts from infected dogs (in bold) and uninfected dogs are shown. Infected = the number of male sand flies attracted to the arm of the Y-tube containing infected odour; uninfected = the number of male sand flies attracted to the arm of the Y-tube containing uninfected odour; no-response = number of sand flies that did not respond in the Y-tube experiment. a = unblinded experimental replicates, b = blinded experimental replicates. Statistical significance was analysed by binomial test comparison of number of sand flies responding to test and control odours (ns = not significant). The experimental outcomes are presented in the same order as for the female bioassays (S1 Table) for ease of comparison. (DOCX) [file ppat.1009354.s002.docx]

| **expt** | **dog** | **Infected**  **odour** | **un-infected odour** | **no-response** | ***P*** |
| --- | --- | --- | --- | --- | --- |
| **1** | **176** vs 021^a^ | 35 | 37 | 8 | *ns* |
| **10** | **141** vs 137^b^ | 34 | 36 | 10 | *ns* |
| **11** | **178** vs 181^b^ | 36 | 36 | 8 | *ns* |
| **6** | **105** vs 037^a^ | 34 | 38 | 8 | *ns* |
| **8** | **140** vs 004^a^ | 37 | 37 | 6 | *ns* |
| **7** | **003** vs 130^a^ | 37 | 36 | 7 | *ns* |
| **5** | **074** vs 093^a^ | 36 | 34 | 10 | *ns* |
| **3** | **082** vs 175^a^ | 38 | 31 | 11 | *ns* |
| **15** | **102** vs 124^b^ | 39 | 32 | 9 | *ns* |
| **13** | **126** vs 169^b^ | 34 | 38 | 8 | *ns* |
| **14** | **047** vs 153^b^ | 35 | 38 | 7 | *ns* |
| **9** | **044** vs 005^a^ | 35 | 38 | 7 | *ns* |
| **2** | **080** vs 136^a^ | 35 | 35 | 10 | *ns* |
| **12** | **134** vs 070^b^ | 38 | 35 | 7 | *ns* |
| **4** | **019** vs 043^a^ | 38 | 35 | 7 | *ns* |
|  | **sum** | **541** | **536** | **123** |  |
|  | **mean±se** | **36.1±0.4** | **35.7±0.6** | **8.2±0.4** |  |
