# Supplementary material for: Odour of domestic dogs infected with Leishmania infantum is attractive to female but not male sand flies: Evidence for parasite manipulation
Source: PLoS Pathog. 2021 Mar 18;17(3):e1009354. doi: 10.1371/journal.ppat.1009354 (PMC7971543; doi:10.1371/journal.ppat.1009354)
Supplement: S3 Table — Dogs were classified as asymptomatic (the absence of clinical signs), oligosymptomatic (the presence of one to three clinical signs) and symptomatic (the presence of more than three clinical signs according to Mancianti et al. [69]. Infection category and symptoms were assessed by Governador Valadares CCZ clinical staff. Parasite load (number of parasites ml-1) was assessed by qPCR as previously described [30]. (DOCX) [file ppat.1009354.s003.docx]

|  | **Female sand fly bioassays** |  | **Male sand fly bioassays** |  |
| --- | --- | --- | --- | --- |
| **Expt.** | dog hair odour pairings | VOC analyser discrimination value | Dog hair odour pairings | VOC analyser discrimination value |
| **1** | **176** vs 021 | **High** vs High | **176** vs 021 | **High** vs High |
| **2** | **141** vs 137 | **High** vs Med | **080** vs 136 | **High** vs Med |
| **3** | **178** vs 181 | **High** vs low | **082** vs 175 | **High** vs low |
| **4** | **105** vs 037 | **Med** vs High | **019** vs 043 | **Med** vs High |
| **5** | **140** vs 004 | **Med** vs Med | **074** vs 093 | **Med** vs Med |
| **6** | **003** vs 130 | **Med** vs Low | **105** vs 037 | **Med** vs Low |
| **7** | **074** vs 093 | **Low** vs High | **003** vs 130 | **Low** vs High |
| **8** | **082** vs 175 | **Low** vs Med | **140** vs 004 | **Low** vs Med |
| **9** | **102** vs 124 | **Low** vs Low | **044** vs 005 | **Low** vs Low |
| **10** | **126** vs 169 | **High** vs High | **141** vs 137 | **High** vs Med |
| **11** | **047** vs 153 | **High** vs Med | **178** vs 181 | **High** vs Low |
| **12** | **044** vs 005 | **Low** vs Low | **134** vs 070 | **High** vs High |
| **13** | **080** vs 136 | **Med** vs Med | **126** vs 169 | **High** vs High |
| **14** | **134** vs 070 | **High** vs High | **047** vs 153 | **Med** vs Med |
| **15** | **019** vs 043 | **Low** vs Low | **102** vs 124 | **Low** vs Low |
