# Supplementary material for: Odour of domestic dogs infected with Leishmania infantum is attractive to female but not male sand flies: Evidence for parasite manipulation
Source: PLoS Pathog. 2021 Mar 18;17(3):e1009354. doi: 10.1371/journal.ppat.1009354 (PMC7971543; doi:10.1371/journal.ppat.1009354)
Supplement: S4 Table — Dog hair odour pairings: the infected and uninfected dog hair odour samples which were compared are shown along with their discrimination values. VOC analyser discrimination value: the VOC analyser discrimination status of each dog is shown. Leishmania infantum infected dogs are denoted in bold text. The infection status of samples 1–9 was known to the experimenter and samples 10–15 were unknown (blinded). (DOCX) [file ppat.1009354.s004.docx]

| **dog number** | **category** | **symptoms** | **parasite load** (parasites mL^-1^) |
| --- | --- | --- | --- |
| 178 | asymptomatic |  | 28.3 |
| 176 | symptomatic | muscular atrophy, weight loss, long nails, conjunctivitis | 50.9 |
| 141 | asymptomatic |  | 13.7 |
| 140 | asymptomatic |  | 40.5 |
| 134 | asymptomatic |  | 50.5 |
| 126 | oligosymptomatic | dermatitis | 853.4 |
| 105 | oligosymptomatic | long nails | 8.5 |
| 102 | asymptomatic |  | 6.9 |
| 082 | asymptomatic |  | 128.0 |
| 080 | symptomatic | dermatitis, long nails, lesions, weight loss | 21.7 |
| 074 | asymptomatic |  | 115.9 |
| 047 | asymptomatic |  | 1.3 |
| 044 | asymptomatic |  | 27.9 |
| 019 | asymptomatic |  | 61.6 |
| 003 | asymptomatic |  | 233.7 |
